# The DIPG/DMG National Tumor Board: The Power of Advocacy

**DOI:** 10.1093/neuped/wuaf009

**Published:** 2025-10-11

**Authors:** Rebecca Ronsley, Greta Peng, Eileen Gutierrez, Patricia Baxter, Jason Fangusaro, Adam L. Green, Lindsay B. Kilburn, Carl Koschmann, Jasia Mahdi, Michelle Monje, Ashley S. Plant-Fox, Nicholas A. Vitanza, Sebastian M. Waszak, Cheng-Chia Fred Wu, Matthew J. Barkovich, David Mirsky, Sabine Mueller

**Affiliations:** 1 Ben Towne Center for Childhood Cancer and Blood Disorders Research, Seattle Children’s Research Institute, Seattle, WA, USA; 2 Department of Pediatrics, Seattle Children’s Hospital, University of Washington, Seattle, WA, USA; 3 Seattle Children’s Therapeutics, Seattle, WA, USA; 4 University of California, San Francisco, San Francisco, CA, United States.; 5 Texas Children’s Hospital, Cancer and Hematology Center, Baylor College of Medicine; 6 Children’s Healthcare of Atlanta and the Aflac Cancer Center, Emory University School of Medicine, Atlanta GA, USA.; 7 Children’s Hospital Colorado, Morgan Adams Foundation Pediatric Brain Tumor Research Program, University of Colorado School of Medicine, Aurora, CO; 8 Center for Cancer and Blood Disorders and The Brain Tumor Institute, Children’s National Hospital, Wasthington DC, USA; 9 Department of Pediatrics, University of Michigan Medical School, Ann Arbor, MI, USA; 10 Department of Neurology and Neurological Sciences, Stanford University, Stanford, CA USA; 11 Ann & Robert H. Lurie Children’s Hospital of Chicago, Northwestern University Feinberg School of Medicine, Chicago, IL, USA; 12 Centre for Molecular Medicine Norway (NCMM), University of Oslo and Oslo University Hospital, Oslo, Norway; 13 Swiss Institute for Experimental Cancer Research, School of Life Sciences, École Polytechnique Fédérale de Lausanne (EPFL), Lausanne, Switzerland; 14 Frailin Biomedical Research Institute, Virginia Tech, VA, USA, Children’s National Hospital Brain Tumor Institute, D.C., USA, and Inova Schar Cancer Institute, VA, USA.; 15 Children’s Hospital of Colorado, Department of Radiology, University of Colorado School of Medicine, Aurora, CO; 16 UCSF Helen Diller Family Comprehensive Cancer Center, United States

**Keywords:** diffuse midline glioma, diffuse intrinsic pontine glioma, pediatric, oncology, advocacy

## Abstract

**Background.:**

Diffuse intrinsic pontine gliomas (DIPG) and diffuse midline gliomas (DMG) are fatal brain tumors and clinical trial enrollment remains a cornerstone of treatment; however, barriers to trial identification, access and enrollment persist. The DIPG/DMG National Brain Tumor Board (DMG NTB) was created based on family foundation advocacy to address these challenges by providing centralized, multidisciplinary guidance to improve access to expertise and facilitate clinical trial awareness and enrollment.

**Methods.:**

We conducted a retrospective descriptive analysis of cases presented to the DMG NTB between November 2022 and December 2024. Data were extracted from standardized intake forms, meeting records and chart reviews, including patient demographics, disease characteristics, molecular findings, referring institution type, trial recommendations, and radiologic evaluations. Geographic reach and provider participation were also analyzed.

**Results.:**

A total of 349 presentations representing 279 unique patients were reviewed. Referrals came from 93 institutions across 35 states, including 48% from treating physicians, 34% via self-referral, and 18% from My DIPG Navigator. The median age was 9 years (range: 1–52), and 83% of patients had undergone tumor biopsy, with molecular profiling available in 81% of those cases. The DMG NTB provided a median of six clinical trial options. Imaging review resulted in additional findings, recommendations, or both in over half of cases. Participation grew by 89.5% over the evaluated time period.

**Conclusions.:**

The DMG NTB has established a scalable, patient-centered system that enhances trial awareness, eligibility screening, care coordination, and multidisciplinary learning for patients with DIPG/DMG. This parent-driven initiative addresses longstanding gaps in access to expertise. Ongoing integration with research networks will be critical to maximizing its impact on outcomes for children and young adults.

## Lay Summary

Diffuse intrinsic pontine gliomas (DIPGs) and diffuse midline gliomas (DMGs) are the most aggressive and deadly brain tumors in children. While new clinical trials are developed based on a better understanding of the biology of these tumors, families may struggle to identify and access these trials. Empowered by patient and family advocates, a group of physicians and scientists focusing on DMG/DIPG came together to help address this gap and created the *DIPG/DMG National Brain Tumor Board (DMG NTB)*. This is a virtual multi-expert review of patient cases from around the country to provide recommendations on diagnosis, imaging, and potential clinical trials. In this analysis we reviewed the 279 patients whose cases were discussed by the DMG NTB between November 2022 and the December 2024. These cases came from 93 different hospitals in 35 states. Families and/or doctors could request help through various paths, including direct referrals and a patient support group called *My DIPG Navigator*. The DMG NTB typically identified an average of six clinical trial options per case, helping families and doctors understand the ones for which their child or patient might qualify. In more than half of the cases, expert review of MRI scans led to new insights or potential changes in care plans. Importantly, participation in the DMG NTB grew steadily over time, showing a strong need for this service. The DMG NTB is helping families and physicians access expert guidance and emerging therapies, while also serving as a valuable educational resource for medical professionals across diverse practice settings. However, broader awareness and accessibility are still needed. The NTB also offers a model that could be adapted to improve care in other rare childhood cancers and diseases.

## Introduction

Diffuse intrinsic pontine glioma (DIPG) and diffuse midline glioma (DMG) are some of the most lethal pediatric central nervous system (CNS) tumors. These gliomas typically arise in midline structures (most commonly the pons), and they are characterized by their highly infiltrative nature, profound treatment resistance, and dismal prognosis. Despite standard-of-care focal radiation therapy, the median overall survival for DIPG remains approximately 11 months and for non-pontine DMG, approximately 13 months, with fewer than 10% of children surviving beyond two years.^1–4^

The landmark discovery of recurrent somatic mutations in genes encoding histone H3 variants, particularly H3K27M, has fundamentally reshaped the biological understanding and classification of these tumors.^5,6^ The 2021 WHO classification now recognizes H3K27-altered diffuse midline glioma as a distinct entity with a universally poor prognosis.^7^ This molecular insight has spurred a surge in biologically informed clinical trial activity aimed at exploiting tumor-specific vulnerabilities.

Therapeutic approaches currently under investigation span a broad spectrum of modalities. Small molecule inhibitors (eg, ONC201, ONC206, and PTC596) have demonstrated CNS penetration and early evidence of clinical activity against H3K27-altered DMG.^8–12^ Similarly, targeted inhibitors of epigenetic regulators (eg, EZH2, HDACs), receptor tyrosine kinases (eg, panobinostat, paxalisib, avapritinib), and cell cycle proteins (eg, ribociclib) are under active clinical investigation.^13–15^ Immunotherapeutic strategies for DIPG/DMG have gained momentum recently, with multiple early-phase trials evaluating chimeric antigen receptor (CAR) T cells targeting GD2, B7-H3, and HER2 antigens.^16–20^ These trials have shown preliminary safety and feasibility in both intravenous and intracranial administration, with evidence of immune activation, and radiographic and clinical responses in some trials.^16–18,21,22^ Oncolytic viruses have emerged as a promising therapeutic strategy for diffuse midline gliomas (DMG), with recent data from a first-in-human trial demonstrating the feasibility and potential clinical activity of intratumoral DNX-2401 administration in children with newly diagnosed DMG.^23^ Furthermore, other immunotherapeutic strategies such as vaccine therapy, and blood brain barrier disruption using focused ultrasound (FUS) are under investigations in clinical trials for DIPG/DMG.^11,24^ DIPG/DMG is strongly influenced by the activity of glutamatergic, GABAergic, and cholinergic neurons,^25–27^ and a growing body of preclinical research has identified potential therapeutic strategies that target these neural pathways, with clinical trials in development. In addition, the use of low intensity FUS to either enhance drug activity in the setting of 5-ALA^28^ or open the blood brain barrier (BBB) to improve drug delivery (eg, doxorubicin, panobinostat, or etoposide) are being evaluated. The spectrum of available and emerging therapeutic options (Table 1) reflects the unprecedented pace of research in this previously stagnant field.

While this surge in clinical trial development offers renewed hope, it also presents profound challenges for families and providers. Identifying and accessing potential trials requires nuanced understanding of eligibility criteria, institutional access, and timing. These are all barriers that disproportionately affect patients outside major academic centers. These challenges are compounded by the urgency with which decisions must be made, often in the weeks immediately following diagnosis, radiation or progression.

In response to this gap, the DIPG/DMG National Brain Tumor Board (DMG NTB) was launched as a centralized, expert resource. Founded through a unique collaboration between parent advocacy groups and pediatric neuro-oncology leaders, the DMG NTB provides real-time multidisciplinary consultation on any newly diagnosed or recurrent DIPG/DMG case across the United States. It convenes experts in neuro-oncology, radiation oncology, neuro-radiology, neurology, neurosurgery, pathology, genomics, and translational research to review cases and guide trial selection and management. The DMG NTB aims to democratize access to cutting-edge science and clinical trials, reduce disparities in care, and support providers and families facing one of the most complex challenges in pediatric oncology as well as to serve as an educational resource for providers and trainees less familiar with DIPG/DMG.

This manuscript reviews the epidemiologic burden of DIPG and DMG, highlights the landscape of emerging therapeutic strategies, and describes the formation, implementation, and early utilization of the DMG NTB as a novel, advocacy-initiated model for centralized expert guidance in the precision medicine era.

## Materials and methods

### The DIPG/DMG National Tumor Board (DMG NTB)

**Figure 1.**
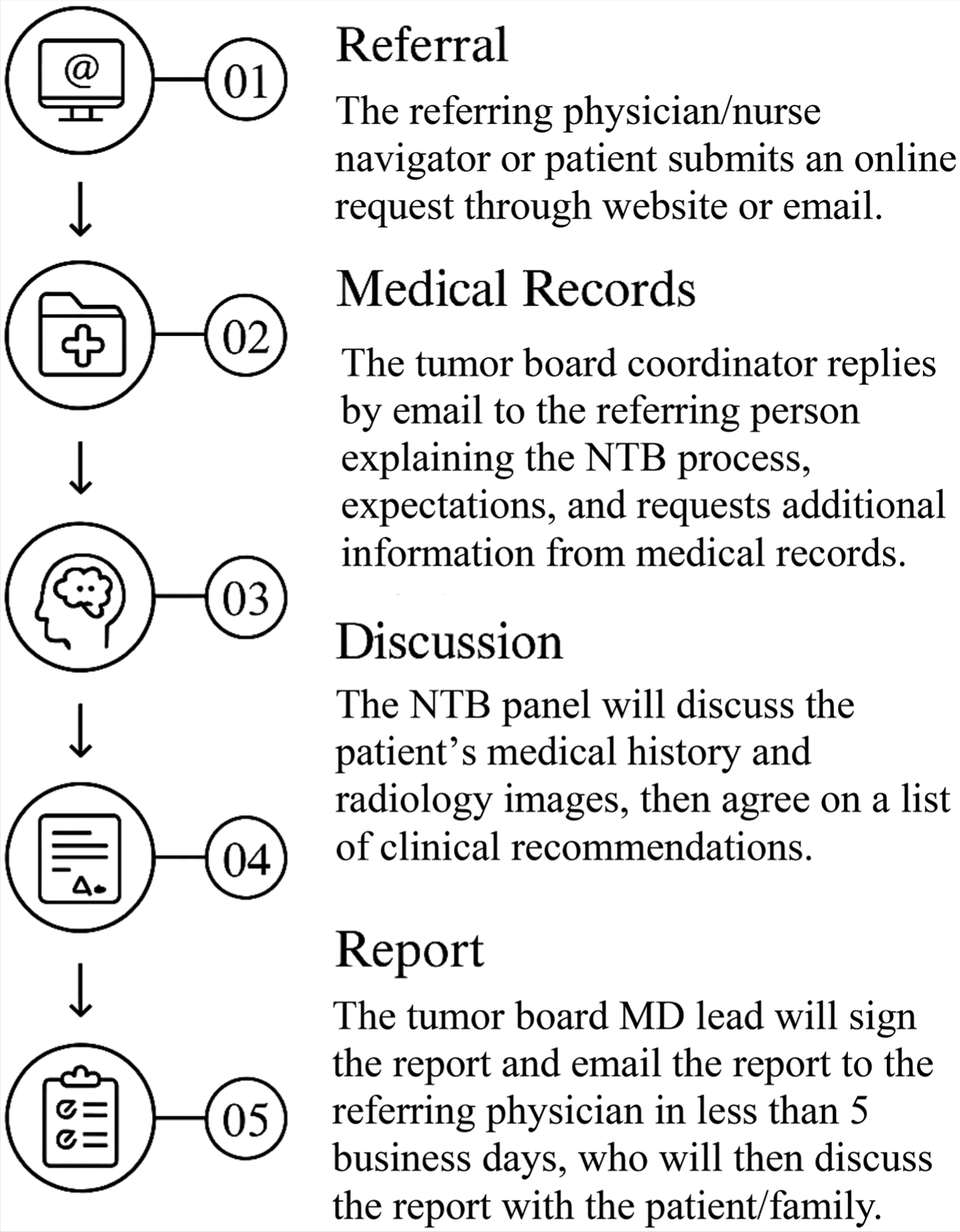
Tumor board flow. Schematic overview of the DMG National Tumor Board (NTB) workflow, including referral, case submission, multidisciplinary review, and dissemination of recommendations to participating clinicians and institutions.

The DMG NTB is a virtual, multidisciplinary consultation forum that meets twice monthly to provide centralized expert input on the diagnosis and management of children and young adults with DIPG and DMG (see https://dmgnationaltumorboard.org). Advocated for by patient families expressing a need for structured support, the DMG NTB was developed to help navigate the increasingly complex clinical trial landscape, streamline the process of assessing potential clinical trial eligibility, and offer a collaborative platform for best practice discussion, imaging review, molecular review, and provider education. Core DMG NTB members rotate in moderating the DMG NTB and are required to attend a minimum of 70% of tumor boards and participate in discussion of each patient. Flow of patients through the DMG NTB is shown in Figure 1. Patients across the United States can be referred through multiple pathways, including treating physicians, the My DIPG Navigator program, or direct family self-referral. The DMG NTB includes pediatric neuro-oncologists, radiation oncologists, neuropathologists, neuroradiologists, genome scientists and translational scientists/clinical trialists from the Pediatric Neuro-Oncology Consortium (PNOC), Collaborative Network for Neuro-oncology Clinical Trials (CONNECT), Children’s Oncology Group (COG) Pediatric Early Phase Clinical Trial Network (PEP-CTN), and Pediatric Brain Tumor Consortium (PBTC) as well as institutions leading single-center clinical trials focused on DMG/DIPG. In patients for whom molecular analysis has not been conducted on the tumor sample, molecular analysis is offered free of charge (MiOncoSeq^29,30^). While not a replacement for tissue but due to ongoing work in the field of liquid biopsy, CSF analysis for cell-free DNA was also offered as an investigational tool to identify genomic information in certain clinical contexts.^31,32^ The DMG NTB aims to ensure equitable access to multidisciplinary expertise and personalized treatment recommendations, focusing on clinical trial options for all patients treated at any center within the United States of America.

For each patient presented, a comprehensive report is generated that includes a summary of clinical history, radiologic review (including restaging), available molecular results, and a detailed assessment of potential clinical trial options with contact information for each trial. These reports are shared directly with the treating team to support shared decision-making with the patient at the local level. Participation of the primary provider or representative is strongly encouraged during tumor board meetings to facilitate real-time discussion, enhance collaborative care, and ensure alignment of expert recommendations with patient-specific context and treatment goals.

### Study design and data collection

This is a retrospective analysis of all patient cases submitted to the DMG NTB from November 2022 to December 2024. Data were obtained from the intake DMG NTB forms completed by the patient’s provider prior to tumor board presentation as well as from meeting records documenting case discussions and corresponding tumor board reports. Additional sociodemographic data was obtained from the intake form and via provided records/chart review. Data collection included demographic and geographic variables such as patient age at the time of presentation, treating institution, city, state, and whether the referring center was academic or community-based. Clinical information encompassed tumor type and location (eg, pontine, thalamic, spinal), time from initial diagnosis, and relapse status (eg, newly diagnosed, progressive, or recurrent disease). Staging and imaging data were abstracted from radiographic summaries, including indicators of disease spread, mass effect, hydrocephalus, and other pertinent findings. Histopathologic and molecular data included histologic diagnosis, biopsy status, H3K27M mutation and histone variant status, and any available molecular or next-generation sequencing results. Information on tumor board participation was also collected, including the number and types of providers present during the discussion (eg, neuro-oncologists, radiation oncologists, neurosurgeons) and the institutional affiliation of the submitting provider. Involvement of the DIPG/DMG nurse navigators was also collected. Finally, tumor board reports were reviewed by the Tumor Board lead of that particular session to determine whether clinical trial options, supportive care strategies, or guidance on surgical or radiation planning were recommended.

### Data analysis

Descriptive statistics were used to summarize categorical variables (eg, sex, relapse status, biopsy status, mutation status) and continuous variables (eg, age). Geographic distribution was visualized using choropleth mapping to display state-level referral patterns. The proportion of cases for which clinical trial options were discussed was calculated, along with the number of unique trials suggested across all reviewed cases. Provider participation rates and disciplinary diversity were also summarized per meeting. All analyses were conducted using SPSS Statistics Version 26.

## Results

**Figure 2.**
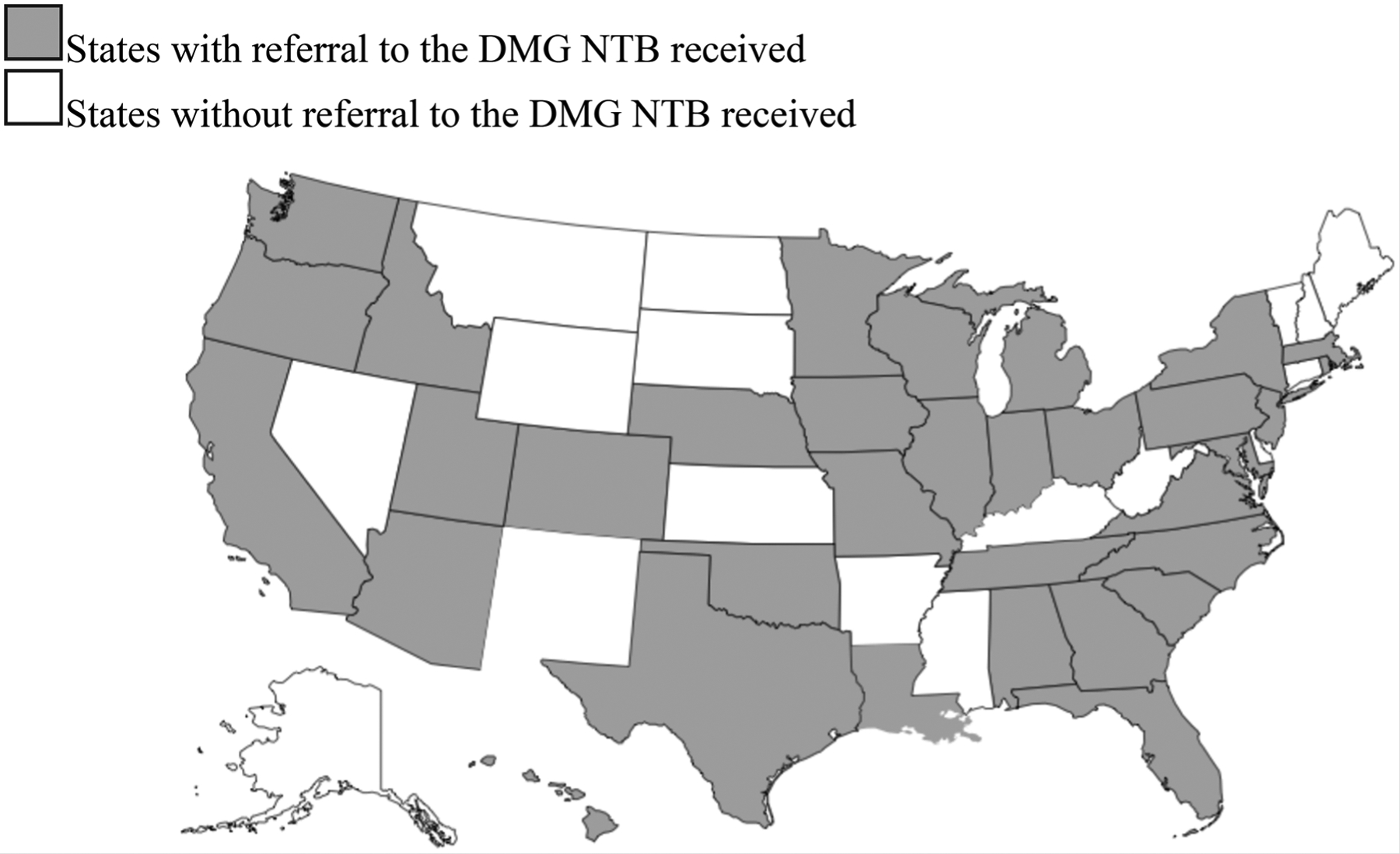
Referral pattern by state. Geographic distribution of NTB case referrals, illustrating the number and origin of cases submitted by U.S. state.

Between November 2022 and December 2024, the DMG NTB reviewed 349 presentations representing 279 unique patients, with 56 individuals presented more than once. The DMG NTB demonstrated broad national reach, receiving referrals from 93 institutions across 35 states (Figure 2). The treating provider was present in 79% of case presentations. Referrals were initiated by treating physicians (48%), self-referral (34%) or My DIPG Navigator (18%).

### Demographic and clinical characteristics

Patient demographics for cases submitted are shown in Table 2. The median age at presentation was 9 years (range 1–52 years), with 261 pediatric patients (up to 14 years old), 82 adolescents and young adults (AYA) (>14 to 39 years old), and 6 adults (≥40 years old). Of the 249 unique patients with available sex data, 52.4% were female and 47.0% were male. Prior therapy is summarized in Table 3. Most patients (88.9%, *n* = 223) received upfront radiation therapy, with 56.8% receiving photon, 9.4% proton therapy, and the remainder unknown radiation therapy type. Eleven percent of patients who received upfront radiation therapy were presented prior to receiving radiation therapy. Concurrent chemotherapy with radiation therapy was administered to 22.6% (*n* = 79), most commonly temozolomide (57.0%), or selinexor as part of trial (Children’s Oncology Group ADVL1414) participation (11.4%). A minority (12.6%, *n* = 44) were enrolled on clinical trials prior to DMG NTB presentation. The most cited individual agents administered after radiation were dexamethasone (69%), bevacizumab (25%), ONC201, and levetiracetam (each 8.3%). Re-irradiation was documented in 33% (56/170) of patients with available follow-up data. Disseminated disease was reported in 15.2% (*n* = 53), involving the leptomeninges (41%), spine (33%), brain (21%), or bone (3%) (including vertebrae, pelvis and long-bones).

### Molecular and histologic profiling

A majority of patients (83.2%, 232/279) had undergone biopsy, and of these, 81.0% (188/232) had molecular profiling available at the time of DMG NTB discussion. Among the remaining, profiling was pending (*n* = 20), not pursued (*n* = 12), or limited by tissue availability (*n* = 12). For those without profiling completed or pending prior to tumor board presentation, 50% ultimately had tumor profiling completed after tumor board presentation, with all but one case completed through analysis offered within the DMG NTB report. Somatic drivers identified included *H3F3A* (97%), *TP53* (48.4%), *ATRX* (18.6%), *PIK3CA* (14.9%), *PPM1D* (13.3%), *NF1* (9.6%), *ACVR1* (10.1%), *PDGFRA* (9.6%), *PIK3R1* (7.9%), *EGFR* (7.4%), *PTEN* (6.4%), *BCOR* (4.3%), and *BRAF* (1.6%), with most patients’ tumors (85.1%) expressing more than one alteration. Germline testing was reported in 16.4% of patients (*n* = 31), with pathogenic variants identified in *APC*, *MSH3*, and *SDHB*.

### DMG NTB recommendations and impact

In all cases, the diagnosis of DIPG/DMG was reviewed and in 97% of cases, the DMG NTB agreed with the diagnosis of DIPG or DMG. Radiologic re-evaluation contributed to revised interpretations or new findings in a subset of patients. Recommendations for additional imaging modalities or sequences were provided in 55.9% and 38.2% of applicable cases, respectively. Imaging recommendations were common: 4.3% required repeat MRIs with protocols to clarify ambiguous lesions, 9.7% needed additional imaging, 55.9% were advised to obtain complete neuro-axis MRI (eg, adding an MRI spine when only an MRI brain was completed), and 38.2% were advised for additional brain sequences (eg, when significant gaps were observed within MRI images or when FLAIR/T2 imaging was missing). When tissue diagnosis and molecular testing were not pursued due to patient or clinician preference, liquid biopsy was offered as a potential avenue to identify molecular alterations. through CSF-based tDNA testing.

Therapeutic agents were discussed for 47.9% of cases (*n* = 167), and many patients had received re-irradiation prior to presentation and additionally, re-irradiation was recommended for consideration in 36% (32/90 patients presented post progression). The DMG NTB provided a median of six clinical trials to consider per patient report (range 0–13), with a median of four trials for which the patient was deemed potentially eligible (range 0–11). Clinical trials recommended as potential options included the following therapies: small molecules, targeted agents, FUS and convection enhanced delivery, oncolytic virus therapy, vaccine therapy, CAR T cell therapy (both intra-cerebroventricular (ICV) and systemic delivery trials), and combination chemotherapy. In 13.7% of patients (34/249) the patient was deemed not eligible for a clinical trial due to functional status and the DMG NTB supported non-tumor directed/supportive care or palliatition/hospice. The DMG NTB report for each patient included potential clinical trial names, contacts, and enrollment information.

### Community engagement and longitudinal data collection

My DIPG Navigator provided support for 177 patients reviewed on the DMG NTB, including 153 prior to DMG NTB review. A workflow is established to consent patients for data sharing through the Children’s Brain Tumor Network (CBTN), though this was only completed in 10% of eligible patients at the time of this analysis.

### Tumor board attendance and growth

Tumor board capacity expanded significantly over the study period, with a 175% increase in cases discussed and an 89.5% increase in total attendance between fiscal years. Attendee roles diversified, with a 300% increase in participation from treating MDs and nurse practitioners (NPs) and a 37.5% increase in core DMG NTB members. Attendance strategies included salary support for core DMG NTB Members, structured reminders, and institutional outreach to broaden participation and educational benefit.

## Discussion

The DMG NTB represents a novel strategy for centralized, multidisciplinary consultation aimed at addressing critical gaps in the care of children and young adults with DIPG and other DMGs. This resource provides high-level, expert access for any patient with a DIPG/DMG within the United States, regardless of location or treating institution. Here, we demonstrate that within its first two years, the tumor board supported 279 patients over 90 institutions and across 35 states, and facilitated actionable clinical trial recommendations, radiologic insights, and community resource integration. Collectively, these results illustrate the DMG NTB’s national impact on multidisciplinary care coordination, clinical trial accessibility, radiologic evaluation, and patient/family engagement in DIPG/DMG management.

These findings highlight several important themes. Importantly, the tumor board served a demographically and geographically diverse population, with referrals originating from both academic and community institutions. Notably, over one-third of cases were submitted by families or patient navigators rather than treating physicians, reflecting a growing trend of patient-driven engagement in care coordination and clinical trial access, especially in the landscape of DIPG/DMG. This democratization of access is essential in a disease where timing is critical and disparities in care remain a concern.

Furthermore, the DMG NTB functioned as a centralized knowledge hub, synthesizing rapidly evolving molecular and clinical trial information into practical, individualized recommendations. Despite significant heterogeneity in molecular testing availability, it is important to note that over 80% of patients who underwent biopsy had some form of molecular profiling reviewed at the time of discussion. The tumor board consistently recommended additional molecular profiling through tissue or, when available, liquid biopsy (within CSF) in cases lacking this data, emphasizing its role in promoting precision/individualized medicine. About half of cases were offered targeted agents as a potential therapy option, reflecting the presence of actionable mutations in an individual tumor, and the availability of these medications at the time of case discussion. Clinical trial options to consider were provided in the majority of cases, with a median of four trials where the patient was potentially eligible listed on each report. The DMG NTB did not generally recommend one specific trial over another and provided information about each trial to facilictae shared decision making between the provider and family. This information led to real-time access to curated trial options, complete with enrollment information, which may substantially improve both awareness and enrollment. Prospective tracking is needed to confirm this impact.

Beyond clinical trial options, the centralized radiologic review process yielded clinically meaningful findings in a majority of patients. Recommendations for additional imaging, particularly completion of neuro-axis evaluation and enhanced brain sequences, were common, and in some cases, altered the interpretation of disease extent or progression. This underscores the added value of subspecialty radiology review with specific expertise in DMG/DIPG to interpret complex cases, which may not be readily available at all institutions.

Importantly, the tumor board fostered a robust educational and collaborative environment. We observed substantial growth in attendance, particularly among referring providers and cross-institutional experts. These discussions not only benefitted individual patients but also served as ongoing education for providers and trainees, especially those in low-volume centers. This model of shared expertise aligns with broader goals of improving equity and consistency in care delivery across pediatric oncology.

### Limitations

Despite the strengths described, several limitations warrant discussion. As a retrospective descriptive analysis, we are limited in our ability to assess patient-level outcomes, including changes in survival, quality of life, or actual clinical trial enrollment. The accuracy of clinical and molecular data was dependent on referring documentation, which may vary in completeness. Although efforts have been made to initiate longitudinal data sharing through the Children’s Brain Tumor Network, fewer than 10% of patients have been successfully enrolled to date, underscoring logistical barriers and the need for ongoing infrastructure development. Furthermore, it is important to highlight that the treatment landscape for these tumors is rapidly evolving. While the DMG NTB provided recommendations for potential options and directed families toward available clinical trials, this process did not constitute a formal second opinion, and the tumor board did not require or oversee the implementation of its recommendations in any case. Additionally, the patient population served lacked diversity within some states, highlighting a critical need to expand outreach and engagement strategies to ensure the tumor board equitably reaches the entire population affected by DIPG/DMG. We hope that through ongoing partnership with providers, we can reach a greater population of patients with DIPG/DMG and expand access to the DMG NTB. Lastly, while the DMG NTB is primarily composed of pediatric neuro-oncology experts, a small number of adult patients have been presented. At present, the tumor board does not include dedicated adult neuro-oncology representation. Future iterations of the board may benefit from the inclusion of adult subspecialists or from establishing clearer age-related eligibility criteria, particularly as treatment paradigms and clinical trial eligibility differ between pediatric and adult populations.

### Future directions

Future directions include formal integration with national pediatric cancer registries to enable longitudinal tracking of clinical trial recommendations, enrollment, and outcomes. A priority will be prospective monitoring of how DMG NTB recommendations influence therapeutic decision-making and trial participation, with particular attention to real-world effectiveness and equity of access. Expansion of patient- and provider-reported experience and satisfaction metrics will also be critical to assess the platform’s utility and to guide iterative improvements, as well as planned follow up surveys to assess trainee and provider learning from the DMG NTB. Importantly, promotion of the DMG NTB resource in geographically and socioeconomically underserved regions is essential to reduce disparities in access to subspecialty expertise. Outreach strategies will include partnerships with community oncology programs, inclusion in national advocacy networks, and collaboration with referring institutions that may lack dedicated pediatric neuro-oncology support. Recognizing the diversity of the patient population, future iterations of the DMG NTB will also aim to enhance linguistic accessibility, particularly through the nurse navigator role, by integrating translation services and culturally responsive communication strategies. Finally, as biologically-driven and precision-based treatment paradigms continue to evolve rapidly in diffuse midline glioma and other rare pediatric cancers, centralized expert platforms such as the DIPG/DMG National Tumor Board will play an increasingly vital role in equitably translating innovation into clinical impact. The DMG NTB model may serve as a scalable and adaptable framework for other high-risk pediatric and adult cancers for which treatment decisions are complex, options are limited, and access to multidisciplinary expertise remains uneven.

### Conclusions

The DMG NTB has successfully operationalized a foundation-driven, patient-centered, expert-guided, and scalable system for supporting families and providers confronting one of the most aggressive pediatric brain tumors. Its early implementation suggests a meaningful contribution to national trial awareness and preliminary eligibility screening, care coordination, and multidisciplinary learning in a historically siloed and inequitable space. This parent-advocated initiative has rapidly become a key national resource, underscoring the unmet need for accessible expertise in navigating the increasingly complex therapeutic landscape of DIPG/DMG. Continued support and longitudinal integration with research networks will be essential to fully realize its potential in improving outcomes for children and young adults with DIPG/DMG.

## Figures and Tables

**Table 1.** Ongoing and emerging clinical trial strategies in DIPG/DMG

Therapeutic strategy	Mechanism/target	Example agents/trials	Notes
**Epigenetic modulators**	Target chromatin remodeling and histone methylation	HDACi inhibitors, GSK-J4 (KDM6A/B inhibitor)	Designed for H3K27-altered tumors
**Small molecule therapies**	Tumor metabolism, dopamine receptor D2, mitochondrial stress	ONC201 (DRD2/ClpP agonist), ONC206, PTC596	ONC201, ONC206 and PTC596 with evidence of CNS penetration, Phase I and Phase II studies in process
**Targeted agents**	Based on molecular features (somatic mutations) within a patient's tumor	Examples: Paxalisib (PI3K/mTOR), Tovorafenib (pan-RAF), everolimus (mTOR inhibition), ribociclib (cell cycle inhibition)	Evidence of CNS penetration, Phase I and Phase II studies in progress
**Immunotherapy—CAR T Cells**	Manufactured T cells targeting tumor surface antigens	GD2, B7-H3, HER2, IL-13ra2	Administered intracerebroventricularly or intratumorally, Phase I studies completed or in progress
**Immunotherapy—checkpoint inhibitors**	Anti-PD-1, anti-CTLA-4 antibodies	Nivolumab, Pembrolizumab (in combination with radiation or vaccines)	Monotherapy and combination studies in progress
**Oncolytic viruses**	Tumor-selective viral replication, immune activation	DNX-2401 (Delta-24-RGD), HSV-1 G207	Delivered via convection-enhanced delivery (CED) or subcutaneous injection; Phase I and Phase II studies in progress
**Radiation-based combinations**	Re-irradiation, radiosensitizer pairing	Re-RT + ONC201; radiation + checkpoint inhibitors; selinexor	Strategies to improve efficacy of standard focal radiotherapy; often in progressive setting
**Chemotherapy**	Conventional cytotoxic agents	Example: bevacizumab, irinotecan, temozolomide, additional combinations	Used in monotherapy or combination therapy
**Vaccine/antigen-based therapies**	Induce specific immune responses to tumor neoantigens	H3.3K27M peptide vaccine trials, dendritic cell vaccines	Personalized and non-personalized vaccine therapies under early investigation
**Convection-enhanced delivery (CED)**	Localized delivery bypassing BBB	IL13-PE, DNX-2401, panobinostat via CED	Invasive but allows high drug concentrations directly to tumor tissue. Phase I/II studies in progress
**Focused ultrasound (FUS)**	Localized delivery through disruption of the BBB	Transcranial FUS divided into two modalities: high-intensity focused ultrasound (HIFU) and low-intensity focused ultrasound (LIFU)	Phase I studies in progress

**Table 2.** Patient demographics

	Total
Age, median (range), years	9 (1–52)
Sex (% male)	47
Race (%)	
White	69
Asian	13
Black	10
Other/unknown	8
Preferred language for communication	
English	97
Spanish	1
Other	2
Disease status (%)	
Newly diagnosed	64
Post progression	36
Primary tumor location (%)	
Pons	62
Thalamus	23
Spinal	7
Brainstem (non-pons)	4
Cerebellum	2
Other	2
Metastatic disease (%)	15

**Abbreviations:** Percentage (%)

**Table 3.** Treatment history in referred patients

	Total
Radiation	91
Upfront radiation	88.9
Re-radiation	16
Chemotherapy	22.6
Bevacizumab	
Clinical trial	12.6

## Data Availability

Data presented in this manuscript is available upon request.

## Key points

- The DIPG/DMG National Tumor Board (DMG NTB) has supported 279 patients across 35 states within two years, offering expert radiologic, molecular, and patient case review and clinical trial recommendations.
- Comprehensive reports including clinical history, imaging interpretation, and trial eligibility are shared with treating teams to support treatment and care coordination.
- The DMG NTB has expanded clinical trial awareness and multidisciplinary collaboration, especially for families and providers outside major pediatric academic centers.

## Importance of the study

This report provides the first comprehensive analysis of the DIPG/DMG National Tumor Board (DMG NTB), a novel, advocacy-initiated model designed to improve access to multidisciplinary expertise and clinical trial guidance for patients with diffuse midline glioma. In contrast to prior literature, which has largely focused on institutional approaches or trial outcomes, this work highlights a scalable, national platform that has democratized the process of clinical care guided by molecular and genomic patient information by supporting 279 patients across 35 states in its first two years. By integrating radiologic review, molecular profiling, and expert-curated trial recommendations into actionable reports shared with treating teams, the DMG NTB addresses critical barriers to care in this high-risk population. Findings demonstrate the DMG NTB’s utility in enhancing trial awareness, care coordination, and provider education, particularly in under-resourced settings. This model offers a blueprint for extending expert consultation to other rare pediatric cancers with evolving therapeutic landscapes, and future integration with research networks may enable longitudinal outcome tracking and broader health equity.

## References

[1] Jansen MH, van Vuurden DG, Vandertop WP, Kaspers GJ. Diffuse intrinsic pontine gliomas: a systematic update on clinical trials and biology. Cancer Treat Rev. 2012;38:27–35. 10.1016/j.ctrv.2011.06.007 21764221

[2] Hoffman LM, Veldhuijzen van Zanten SEM, Colditz N, Clinical, radiologic, pathologic, and molecular characteristics of Long-Term survivors of diffuse intrinsic pontine glioma (DIPG): a collaborative report from the international and european society for pediatric oncology DIPG registries. J Clin Oncol. 2018;36:1963–1972. 10.1200/jco.2017.75.9308 29746225 PMC6075859

[3] Cooney T, Lane A, Bartels U, Contemporary survival endpoints: an international diffuse intrinsic pontine glioma registry study. Neuro Oncol. 2017;19:1279–1280. 10.1093/neuonc/nox107 28821206 PMC5570207

[4] Mackay A, Burford A, Carvalho D, Integrated molecular meta-analysis of 1,000 pediatric high-grade and diffuse intrinsic pontine glioma. Cancer Cell. 2017;32:520–537.e5. 10.1016/j.ccell.2017.08.017 28966033 PMC5637314

[5] Schwartzentruber J, Korshunov A, Liu XY, Driver mutations in histone H3.3 and chromatin remodelling genes in paediatric glioblastoma. Nature. 2012;482:226–231. 10.1038/nature10833 22286061

[6] Wu G, Broniscer A, McEachron TA, ; St. Jude Children’s Research Hospital–Washington University Pediatric Cancer Genome Project Somatic histone H3 alterations in pediatric diffuse intrinsic pontine gliomas and non-brainstem glioblastomas. Nat Genet. 2012;44:251–253. 10.1038/ng.1102 22286216 PMC3288377

[7] Louis DN, Perry A, Wesseling P, The 2021 WHO classification of tumors of the central nervous system: a summary. Neuro Oncol. 2021; 23:1231–1251. 10.1093/neuonc/noab106 34185076 PMC8328013

[8] Saratsis AM, Knowles T, Petrovic A, Nazarian J. H3K27M mutant glioma: disease definition and biological underpinnings. Neuro Oncol. 2024;26:S92–s100. 10.1093/neuonc/noad164 37818718 PMC11066930

[9] Chi AS, Tarapore RS, Hall MD, Pediatric and adult H3 K27M-mutant diffuse midline glioma treated with the selective DRD2 antagonist ONC201. J Neurooncol. 2019;145:97–105. 10.1007/s11060-019-03271-3 31456142 PMC7241441

[10] Arrillaga-Romany I, Gardner SL, Odia Y, ONC201 (dordaviprone) in recurrent H3 K27M-Mutant diffuse midline glioma. J Clin Oncol. 2024;42:1542–1552. 10.1200/jco.23.01134 38335473 PMC11095894

[11] Kumar SS, Adile A, Kogiso M, Preclinical studies of BMI-1 modulator PTC596 in diffuse intrinsic pontine gliomas, pediatric high-grade gliomas and medulloblastoma. J Clin Oncol. 2018;36:e14051–e14051. 10.1200/JCO.2018.36.15_suppl.e14051

[12] Venneti S, Kawakibi AR, Ji S, Clinical efficacy of ONC201 in H3K27M-mutant diffuse midline gliomas is driven by disruption of integrated metabolic and epigenetic pathways. Cancer Discov. 2023; 13:2370–2393. 37584601 10.1158/2159-8290.CD-23-0131 PMC10618742

[13] Monje M, Cooney T, Glod J, Phase I trial of panobinostat in children with diffuse intrinsic pontine glioma: a report from the pediatric brain tumor consortium (PBTC-047). Neuro Oncol. 2023;25:2262–2272. 10.1093/neuonc/noad141 37526549 PMC10708931

[14] Hennika T, Hu G, Olaciregui NG, Pre-clinical study of panobinostat in xenograft and genetically engineered murine diffuse intrinsic pontine glioma models. PLoS One. 2017;12:e0169485. 10.1371/journal.pone.0169485 28052119 PMC5215670

[15] Mayr L, Neyazi S, Schwark K, Effective targeting of PDGFRA-altered high-grade glioma with avapritinib. Cancer Cell. 2025;43:740–756. e8. 40086436 10.1016/j.ccell.2025.02.018 PMC12121847

[16] Vitanza NA, Ronsley R, Choe M, Intracerebroventricular B7-H3-targeting CAR T cells for diffuse intrinsic pontine glioma: a phase 1 trial. Nat Med. 2025;31:861–868. 10.1038/s41591-024-03451-3 39775044 PMC11922736

[17] Majzner RG, Ramakrishna S, Yeom KW, GD2-CAR T cell therapy for H3K27M-mutated diffuse midline gliomas. Nature. 2022;603:934–941. 10.1038/s41586-022-04489-4 35130560 PMC8967714

[18] Monje M, Mahdi J, Majzner R, Intravenous and intracranial GD2-CAR T cells for H3K27M(+) diffuse midline gliomas. Nature. 2025;637:708–715. 10.1038/s41586-024-08171-9 39537919 PMC11735388

[19] Gust J, Cole BL, Ronsley R, Locoregional infusion of EGFR806-CAR T cells for recurrent or refractory pediatric CNS tumors: results of the completed BrainChild02 phase 1 clinical trial. Neuro Oncol. 2025; 27:2170–2181. 10.1093/neuonc/noaf064 40070357 PMC12448881

[20] Brown CE, Hibbard JC, Alizadeh D, Locoregional delivery of IL-13Rα2-targeting CAR-T cells in recurrent high-grade glioma: a phase 1 trial. Nat Med. 2024;30:1001–1012. 10.1038/s41591-024-02875-1 38454126 PMC11031404

[21] Ronsley R, Bertrand KC, Song EZ, CAR T cell therapy for pediatric central nervous system tumors: a review of the literature and current North American trials. Cancer Metastasis Rev. 2024;43:1205–1216. 10.1007/s10555-024-10208-4 39251462 PMC11554695

[22] Lin FY, Stuckert A, Tat C, Phase I trial of GD2.CART cells augmented with constitutive interleukin-7 receptor for treatment of high-grade pediatric CNS tumors. J Clin Oncol. 2024;42:2769–2779. 10.1200/jco.23.02019 38771986 PMC11305939

[23] Gállego Pérez-Larraya J, Garcia-Moure M, Labiano S, Oncolytic DNX-2401 virus for pediatric diffuse intrinsic pontine glioma. N Engl J Med. 2022;386:2471–2481. 10.1056/NEJMoa2202028 35767439

[24] Thompson EM, Ashley DM, Ayasoufi K, A peptide vaccine targeting the CMV antigen pp65 in children and young adults with recurrent high-grade glioma and medulloblastoma: a phase 1 trial. Nat Cancer. 2025;6:1559–1569. 10.1038/s43018-025-00998-z 40506525

[25] Venkatesh HS, Johung TB, Caretti V, Neuronal activity promotes glioma growth through neuroligin-3 secretion. Cell. 2015;161:803–816. 10.1016/j.cell.2015.04.012 25913192 PMC4447122

[26] Taylor KR, Barron T, Hui A, Glioma synapses recruit mechanisms of adaptive plasticity. Nature. 2023;623:366–374. 10.1038/s41586-023-06678-1 37914930 PMC10632140

[27] Barron T, Yalçın B, Su M, GABAergic neuron-to-glioma synapses in diffuse midline gliomas. Nature. 2025;639:1060–1068. 10.1038/s41586-024-08579-3 39972132 PMC11946904

[28] Schaab L, Ferry Y, Ozdas M, Exth-49. FOCUSED ultrasound and 5-ala mediated elimination of diffuse midline glioma. Neuro Oncol. 2021;23:vi174–vi174. 10.1093/neuonc/noab196.688

[29] Koschmann C, Wu Y-M, Kumar-Sinha C, Clinically integrated sequencing alters therapy in children and young adults with high-risk glial brain tumors. JCO Precis Oncol. 2018;2:1–34. 10.1200/PO.17.00133 PMC7434092 32832832

[30] Mody RJ, Wu Y-M, Lonigro RJ, Integrative clinical sequencing in the management of refractory or relapsed cancer in youth. JAMA. 2015;314:913–925. 26325560 10.1001/jama.2015.10080 PMC4758114

[31] O'Halloran K, Crotty EE, Christodoulou E, Targeted detection of sequence variants in cell-free DNA from cerebrospinal fluid in pediatric central nervous system tumors. Front Oncol. 2024;14:1513073. 10.3389/fonc.2024.1513073 39834946 PMC11743934

[32] Cantor E, Wierzbicki K, Tarapore RS, Serial H3K27M cell-free tumor DNA (cf-tDNA) tracking predicts ONC201 treatment response and progression in diffuse midline glioma. Neuro Oncol. 2022;24:1366–1374. 10.1093/neuonc/noac030 35137228 PMC9340643

